# The Application of Cold Atmospheric Plasma (CAP) in Barley Processing as an Environmentally Friendly Alternative

**DOI:** 10.3390/foods14091635

**Published:** 2025-05-06

**Authors:** Norman Barner, Michael Nelles, Leif-Alexander Garbe

**Affiliations:** 1 Department of Agriculture and Food Sciences, University of Applied Sciences Neubrandenburg, Brodaer Straße 2, 17033 Neubrandenburg, Germany; garbe@hs-nb.de; 2 Department of Waste and Resource Management, Faculty of Agriculture and Environmental Sciences, University of Rostock, Justus von Liebig Weg 6, 18059 Rostock, Germany; michael.nelles@uni-rostock.de

**Keywords:** barley grains, cold atmospheric plasma, food processing, microbial inactivation, reactive species, mycotoxins, disinfestation, germination, plant growth, detoxification

## Abstract

Cold atmospheric plasma (CAP) is a novel and versatile technology, which is not yet used in the food and agricultural sector for barley processing. In lab-scale applications, the technology shows potential in extending shelf life and ensuring food safety and quality, e.g., during storage. CAP reactive nature counteracts insect pests, fungi, and bacteria, but also improves seed germination and facilitates plant growth not only under stress conditions. Its generation does not require water, chemicals, or solvents and consumes little energy due to low operating temperatures (<60 °C) with a short time span that makes additional production steps (e.g., cooling) obsolete. Therefore, CAP is a sustainable technology capable of further optimising the use of limited resources with the potential of offering solutions for upcoming environmental challenges and political requirements for replacing existing practices and technologies due to the growing impact of climate change. This review summarises recent developments and findings concerning CAP application in barley production and processing with air as the process gas. Furthermore, this comprehensive overview could help identify further research needs to overcome its current technical limitations, e.g., efficiency, capacity, etc., that hamper the upscale and market introduction of this environmentally friendly technology.

## 1. Introduction

Barley (*Hordeum vulgare* L.) is an important crop and staple food, with a production of approximately 145.6 million metric tons globally in 2021 [1]. This commodity is mainly used for feeding animals as well as in the production of food and malt [2], which is an ingredient for beers, distilled spirits, and some baked products [3]. Abiotic stress, insect pests, disease, poor management or production practices, and microorganisms like fungi can lead to losses in barley production [2,3].

The ongoing climate change already had negative effects on yield and production in many of the barley-producing regions worldwide, and approx. 16.1% of its current production might be lost in Western Europe in the future [4]. Changes in weather patterns will likely cause favourable conditions for certain insects and microbiological pathogens [3].

The European Commission (EC) has recognised the emerging climate change and ongoing environmental degradation as existential threats to our planet and launched the European Green Deal in December 2019. The goal is to transform all sectors of the economy to make them more resource-efficient, resilient, and competitive while offering fairer economic gains [5]. This initiative includes the Farm to Fork Strategy (F2F), which specifies the goals for the food and agricultural sector. As the second largest emitter (11%) of greenhouse gas emissions within the EU [6], it consumes large amounts of natural resources and causes biodiversity loss [7]. Therefore, the EC pledged to reduce the use of chemical and hazardous pesticides by 50% (as defined in Annex II to Regulation (EC) No 1107/2009 [8]) and to organically farm a quarter of the agricultural land until 2030 [9]. 

However, critics argue that this policy is going to lead to a significant decline in production (e.g., by 20% for cereals) and a price increase within the EU for agricultural commodities (by 12.5% in the case of cereals) and food [10]. Furthermore, the EU might no longer export cereals and lose its self-sufficiency [10]. Thus, innovation is instrumental for the EC to overcome barriers to reach its ambitious goals [7]. This underlines the demand for innovative solutions like cold atmospheric plasma (CAP), which are sustainable, affordable, less invasive, and chemical-free alternatives to existing practices.

CAP, as a physical technology, can potentially replace or reduce the demand for agricultural chemicals like fertilisers and pesticides due to its detrimental effect on pests [11] and stimulating capability on germination and plant growth [12]. Therefore, it could be considered a green technology [11] also qualifying for organic farming. Its application does not require using chemicals, solvents, or water, therefore leaving no harmful pollutants, and it operates at low temperatures with a short time span (<60 °C), thus consuming little energy [11,13]. Thus, CAP technology saves water and reduces costs and the necessity for (waste) water management and cooling systems, as water scarcity might be a rising problem with climate change [14]. Therefore, CAP could ensure higher yields while saving resources and at least partially lower the burden on nature and the environment.

CAP mainly affects the surface and does not alter internal quality product parameters [11]. Its reactive species disinfest cereal grains and decompose quickly after treatment without leaving harmful residues [12]. This attribute can ensure crop quality and quantity during storage necessary for providing sufficient food in the future, as the UN has projected a global population of 10.4 billion in 2100 [15].

This review summarises the latest developments and findings in CAP treatment using Dielectric Barrier Discharge (DBD) systems and air as the process gas for seed germination, plant growth, disinfestation, and bacterial and fungal decontamination, especially in barley grains. Those findings match the main objectives of the project “Physics for Food and Feed”, which aims at further developing and optimising CAP applications for practical use in the food and agricultural sector [16,17,18]. Consequently, the literature was reviewed and further research needs were identified.

## 2. The Basics of Plasma

**Figure 1:**
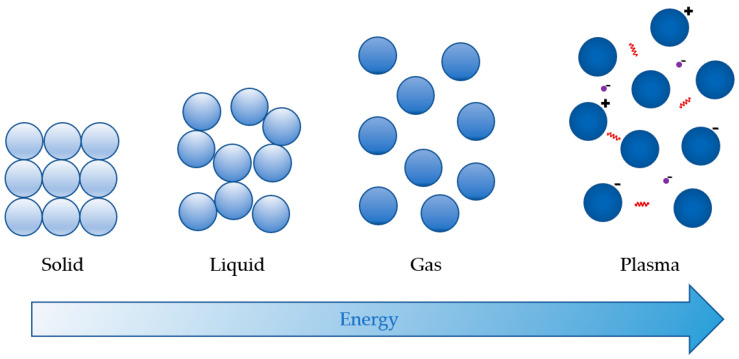
The four states of matter (adapted from [22]).

Plasma is an ionised gas, i.e., the fourth state of matter that is distinct from solids, liquids, and gases, as indicated in Figure 1 below. It can be generated using rising temperatures or electric discharge as an energy source [19], but also by magnetic fields, microwaves, or radio-frequencies [20]. Due to their low mass and high mobility, electrons capture energy first and subsequently transmit it to all other plasma components, delivering energy for plasma-chemical processes like ionisation, excitation, dissociation, etc. [21]. Therefore, plasma is comprised of excited (charged) atomic, molecular, ionic and radical species, free electrons, molecules in the fundamental state, and electromagnetic radiation (UV photons and visible light) [11]. The type of reactive species and plasma energy is determined by the strength of plasma, generating energy source [20]. It naturally appears on Earth in the form of aurorae (northern or southern lights) and lightning [21].

Plasmas can be classified as thermal (equilibrium) and nonthermal (nonequilibrium) plasma [11,23]. In the equilibrium state, electrons, ions, and neutrals of the plasma have roughly the same temperature and energy that result in a high overall temperature (~10,000 K) and high degree of ionisation (~100%) [11]. On the contrary, plasma in the nonequilibrium state has electrons and ions at different temperatures and a low degree of ionisation (below 1%) [11]. Often, the electron temperature is about 10,000 K, but the gas temperature is close to ambient [21]. This category also covers cold plasma with temperatures not exceeding 60 °C [23,24], which mitigates the risk of damaging food and agricultural products by heat.

**Figure 2:**
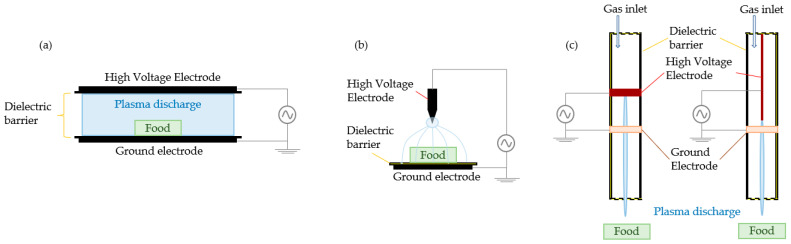
Different CAP systems: (**a**) dielectric barrier discharge, (**b**) corona discharge, (**c**) plasma jets (Adapted from [12,26]).

CAP generation for food processing often uses DBD systems [23,24], but plasma jets, corona discharge, radio-frequency and microwave discharge can also be found [13,25]. The first three mentioned systems are briefly explained below and illustrated in Figure 2.

### 2.1. Dielectric Barrier Discharge (DBD)

DBD systems can be built planar or cylindrical by placing two electrodes parallel to one another, one current-carrying and the other one grounded, with a gap between where the process gas will be ionised. A dielectric (insulator) material has to be placed between the electrodes to avoid sparks [11,12,24].

Its advantages are flexible operating parameters (gas, flow rate, pressure, etc.), cost, safety, reliability, power supply system, design, and simple, robust configuration [11,24,26]. The limitation of the electrode gap size to a maximum of several centimetres is a disadvantage for direct sample treatment. An increasing electrode gap increases the required electric field and voltage to generate plasma [27]. Discharges are often not uniform and characterised by countless microdischarges [21]. The applicable electric parameters vary depending on the process gas and the electrode gap size [28,29].

There are different setups, such as a Surface Dielectric Barrier Discharge (SDBD), basically consisting of two electrodes placed on both sides of an insulator [30], which is often used as an indirect contact system [25]. In a Diffuse Coplanar Surface Barrier Discharge (DCSBD) system, strip-like electrodes are built into in insulator board. This setup for direct treatment has benefits regarding energy consumption and exposure time [31]. Cascaded dielectric barrier discharge (CDBD) is a combination of a UV-emitting excimer and a DBD system, improving the microbicidal effect due to a more uniform discharge and the formation of more reactive species compared to standard DBD [32].

### 2.2. Corona Discharge (CD)

Corona discharges require two electrodes with different geometries, where one has a smaller curvature radius, like a sharp point, edge, or thin wire [21]. The occurring electric field ionises the surrounding gas with a crown-shaped luminance, also known as “corona” [27]. Corona discharge systems work with high voltages and are easy to operate at low gas temperatures and low power [11,24]. They can only treat small areas and produce inhomogeneous discharge when applied directly [11]. However, it is also possible to build CD in an array system, i.e., placing several pin electrodes on a plate, thereby generating a more homogeneous discharge and treating a larger area [33].

### 2.3. Plasma Jet (PJ)

Plasma jets can be built using an annular DBD system with cylindrical electrodes and dielectrics forming the discharge gap able to treat a large quantity or fast flow of gas [24]. But they can also use a corona discharge setup, i.e., an internal needle or pin surrounded by the ring-shaped ground electrode [22]. Plasma jets are, in comparison with DBD, more localised and intense due to turbulent gas flow [34], but they are not limited to the sample size [24]. Larger areas can be treated by movable single jet or multijet array installations [12,27]. Array setups enable using different process gases at each jet [27], therefore changing the gas chemistry, reactive species density, and gas kinetics [12]. The generated plasma is homogeneous, uniform, and stable [35]. But plasma jet systems can be costly, especially when operating with noble gases [11] and high gas flow rates [22].

### 2.4. Plasma Chemistry

Plasma chemistry relies on the chemical composition of the process gas and its flow rate, humidity, electrode material, applied voltage, frequency, and power [11,24]. The formation rate of reactive plasma species increases with increased energy input [36]. Process gases like air, oxygen, nitrogen, and noble gases, or mixtures thereof, are used for the CAP treatment of food and agriculture products [23,24,37,38,39]. Ionising noble gases is easier, requires less voltage, and generates a more uniform discharge [21]. Due to lower concentrations of reactive species, they are often mixed with oxygen or air [24]. The flow rate sets the gas residence time in the discharge area and thus determines the concentration of reactive species. A closed system with no gas flow will offer the highest density of radicals due to accumulation, whereas during a steady gas flow, constant generation of reactive species with a lower concentration occurs [39].

**Figure 3:**
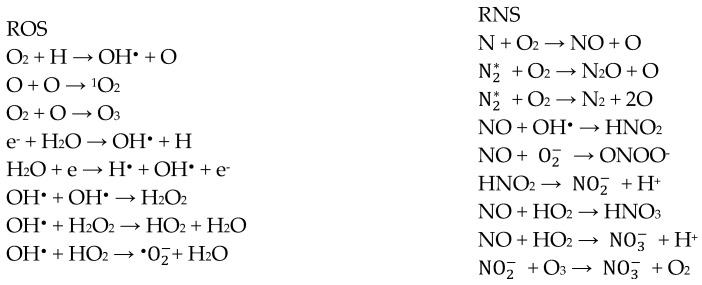
Possible pathways of ROS and RNS formation while using ambient air as process gas [28,36,42,44].

Using ambient air as process gas still has great advantages compared to other gases due to its availability, cost, and presence of oxygen [11]. However, air provides more reactive oxygen species (ROS) than reactive nitrogen species (RNS) when low discharge power is applied. However, the opposite occurs when higher discharge power is applied [36]. Figure 3 below shows possible reactions for forming ROS and RNS from ambient air. This can include hydroxyl radicals, nitrogen oxides, nitrous oxides, ionic nitrogen, peroxides, atomic oxygen, ozone, etc. [11,12,24]. Interactions with air humidity and/or moisture on the product notably affect plasma physics and chemistry, forming a great range of compounds [40], which mainly includes OH^•^ but also H_2_O_2_, HO_2_, HNO_x_, etc. [12]. An increasing humidity impedes the transmissibility of UV radiation, thereby reducing plasma homogeneity [24,32]. It also requires higher voltage for ionisation and decreases O_3_ concentration due to forming nitrogen oxides with higher oxidation levels like N_2_O_3_ and N_2_O_5_ [25]. More than 75 unique chemical species were identified in air plasma with a wide range of half-life periods involving 500 simultaneous reactions [41]. The half-life of the reactive species differs, for example, 1 ms for hydrogen peroxide, 1 µs for singlet oxygen, and 1 ns for hydroxyl radicals, which restricts their penetration depths [40,42]. The decay is determined by its reactions with surfaces, temperature, relative humidity, and concentration [43]. The surface of a solid product can only be penetrated a few micrometres depending on water content and porosity. However, they might be desorbed rebounding into the gas phase if no reaction with the product occurs [40].

In general, gas mixes containing oxygen are desirable irrespective of the plasma source and configuration, forming highly reactive compounds like atomic oxygen, excited oxygen molecules, ozone, etc., that show strong antimicrobial and insecticidal effects due to their potent oxidising capabilities [11,24]. They can also oxidise chemical compounds in food [45], leading to colour changes [25] or causing lipid oxidation in high-fat products [13], forming off-flavours. Corrosion damage to machinery is possible too [22]. Controlling plasma chemistry is key for ensuring a constant desired CAP effect, but difficult to achieve due to fluctuating air humidity and possible emission of moisture from products [13].

Direct plasma treatment includes exposure to luminescence (UV and visible light) [11] and short-living reactive species, enabling the highest energy transfer and bombardment with energetic ions. Those synergy effects are missing during indirect treatment, making it often less effective due to the remote location of the plasma source [11,37]. However, during longer indirect exposure (e.g., in-package treatment), indirect application might also be more effective [39]. With growing distance to the plasma source, the concentration of ROS increases while the concentration of RNS decreases [29]. Less heat is transmitted during indirect application [24], which allows higher ionisation energy, resulting in increased ionisation with hotter and more reactive plasma species [29].

## 3. The Potential of CAP in Food and Agriculture

**Figure 4:**
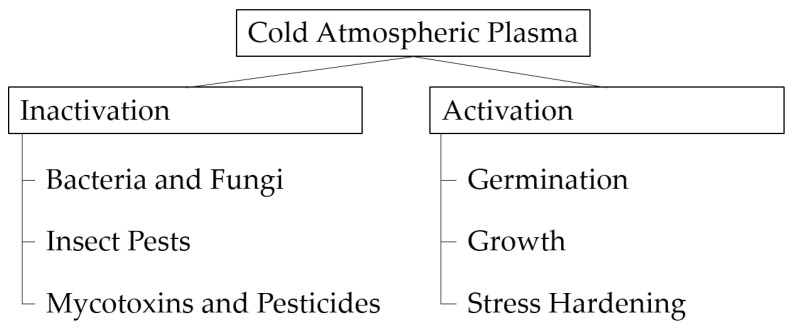
Potential CAP use in different production stages.

CAP treatment can be used for plenty of different applications. It facilitates germination [46] and growth in the early stage of plant development [23,47], even under stress conditions [48,49]. Furthermore, CAP can inactivate fungi [50], bacteria [51], or insects [52] and, at the same time, decompose mycotoxins [53] and pesticides present on the grains [12]. It shows potential in reducing the required amount of fungicides due to synergetic effects [47]. The Figure 4 below shows the potential CAP use in different production stages.

A significant advantage is its production on-site and on demand without the use of water or solvent [13]. CAP effects are limited to the product surface not affecting internal food quality characteristics [34]. Compared with other surface methods like UV radiation, plasma flows around solid surfaces (indirect treatment) or is generated on all sides of the treated barley grains (direct application), which eliminates shadow effects and makes the process more effective [54]. This cold technology can make certain process steps obsolete (e.g., heating or cooling during decontamination), thereby improving process efficiency and speed [26]. Due to the fast decay of the plasma species, no active reactive species are expected to be on the product when it reaches the customer [24].

Thus, CAP could meet consumer demand for high quality, minimally processed and clean labelled products with fewer chemicals, which are associated with healthiness and environmental sustainability [11,13,24]. This can drive innovation in the food and agricultural industry. Studies on consumer acceptance of CAP-treated barley products are missing at the moment, which is necessary to evaluate their whole potential for the industry.

Until now, little research has been conducted on the safety of plasma-treated foods, so the knowledge about mutagenic or cytotoxic effects is limited. Peroxidation of lipids can form toxic aldehydes [55], and the fixation of atmospheric nitrogen can leave nitrite and nitrate on products [56]. However, this precipitation was not always observed [57], potentially due to different CAP systems. Reactions of CAP with the food matrix might form new, unknown, toxic, or mutagen substances. However, CAP has no genotoxic effects on grains of barley [58] or wheat [12,59]. However, different in vitro tests using mammalian cells showed a mixed picture with regard to acute cytotoxicity and mutagenic effects [57,60]. Nevertheless, feeding tests of different CAP-treated products in rodents and insects had no adverse effects [60,61,62]. Thus, CAP-treated products are most likely safe when ingested.

**Figure 5:**
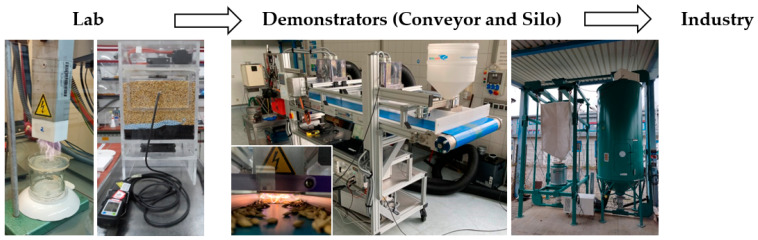
Vision of the Project “Physics for Food” (pictures 3 and 4 reproduced with permission from Paulina Druse, INP Greifswald, 2022).

Different projects currently work on industrial implementation. For example, “Physics for Food” optimises a conveyor belt system and silo demonstrator for inactivating microbiological and insect pests on barley grains before or during storage [16,17,18]. The vision can be seen in the Figure 5 below. The conveyor belt is equipped with four consecutively installed plasma sources, allowing direct and continuous treatment [17]. The barley grains in the silo are fumigated with air plasma, i.e., using an indirect system. Both applications can reduce the carbon footprint by minimising storage losses, finally resulting in decreasing the use of pesticides and fertilisers due to higher crop output [16]. This addresses the main goals of the European Green Deal and the related F2F strategy.

Other researchers work on a fluidised-bed dryer with an integrated plasma system, which allows optimisation of the exposure time to the plasma without extending the entire processing time [12,59]. This approach shows the potential of utilising synergistic effects by incorporating CAP into existing processes for optimising food safety and quality.

For industrial-scale CAP application, fast and continuous treatment of large volumes or areas is key to success in the food and agriculture sector. Different projects [59,63] demonstrate that these issues can be mastered. Thus, more market and business-oriented solutions using this technology can be expected in the near future.

## 4. CAP Effects on Microorganisms

Barley grains can be contaminated with different microorganisms, e.g., in the field, at storage, and during further processing [35]. Bacteria and fungi, including their metabolites, are more detrimental than other microorganisms [64]. They lead to severe economic and quality losses due to spoilage. Optimal storing conditions, good manufacturing, and good agricultural practices can help limit contaminations and spoilage but are not always effective in eliminating them [64]. Chemical grain treatment is frequently applied because of its high efficiency and affordability, but it compromises ecosystems, leaves residue, and impairs product quality [65]. The EU’s plan to move away from chemicals and find less invasive, efficient, and ecological alternatives could be the chance for CAP technology. This section will focus on the overall effects of CAP on microorganisms present in barley.

*Temperature*

The low temperatures of CAP (often not exceeding 60 °C), and the thermal effects on microbiological inactivation and the product itself should be negligible [24]. During conventional heat treatment, inactivation starts between 60 to 80 °C, but some microorganisms and most spores can survive [66].

*Reactive species, UV light, and charged particles*

The inactivation process by air plasma starts with reactive species etching and eroding the microorganisms’ surface, leading to damage to the cell membrane and wall [24]. ROS endanger cellular viability by lipid peroxidation in cell membranes, altering its structure and permeability [67]. The following cell deformation, possible cell leakage, and fragmentation of cellular proteins, including DNA, can finally cause cell death [23,24]. Moreover, reactive species can migrate into the cells, decreasing their pH [20]. This can lead to inactivation if the cell is not able to counterbalance this change [68].

During direct CAP treatment, UV radiation can play a crucial role in microbiological inactivation [69]. DNA can be damaged by high-energy UV photons emitted from excited particles, with the strongest biocidal effects ranging from 220 to 280 nm [70]. The maximum UV absorption of DNA lies in the UV-C spectrum at 254 nm [29] or at 260 nm [71]. UV-B shows less impact, and UV-A even has a minor impact on microorganisms [72]. However, UV radiation can be absorbed by present water vapour, mitigating its effectiveness [67]. UV photons can form reactive species like ozone and unleash a synergistic effect, enhancing the antimicrobial properties of the treatment [22,32,45].

**Figure 6:**
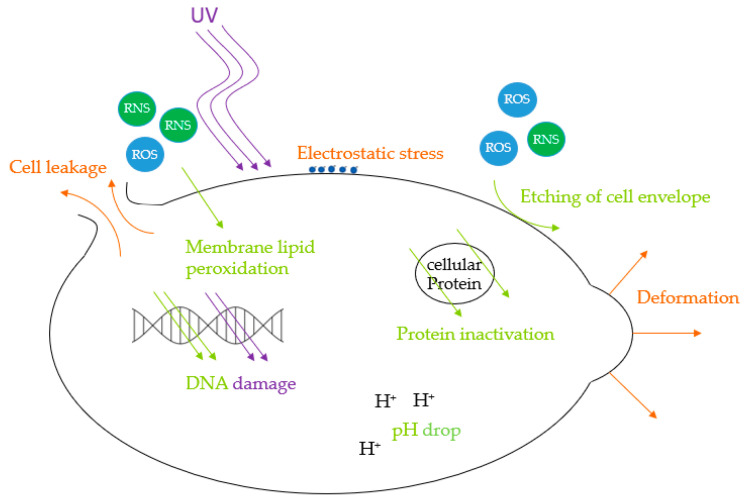
Mechanisms of antimicrobial CAP effects.

Furthermore, during direct exposure, charged plasma particles can accumulate on the surface, leading to electrostatic stress, which can exceed the tensile strength of the cell membrane, causing morphology deformation. This can perforate the cell envelope and lead to enhanced migration of reactive species into the cell with further damage [73]. The injuries caused can exceed the ability of the cellular repair mechanism [67], which can cause cell death [23] or significantly reduce metabolic activity, i.e., leaving the microorganisms in a viable-but-nonculturable state [74]. The Figure 6 below illustrates antimicrobial CAP effects.

*Humidity*

The antimicrobial effects are increased by the humidity of the process gas [22] due to the production of OH^•^ radicals and others [24,73]. Depending on the plasma sources, sample, and microorganisms, optimal humidity can be achieved before a further increase reduces the microbiological inactivation again [75]. The latter is caused by a quenching effect weakening the plasma due to electron–molecule collisions leading to energy losses [75]. Higher humidity causes reduced plasma homogeneity, poorer UV radiation transmissibility (during direct treatment), and microbial cells protected by water films [24,75]. The optimal humidity for inactivating an oxidation-sensitive microorganism can hamper the reduction of a species vulnerable to UV radiation if CAP is applied to mixed microflora [32]. This shows the significance of choosing and applying the optimal value for relative humidity to maximise the inactivation of the main pathogenic organism. The gas humidity can also be affected by the water content of a product [24,75] or influenced by other processing steps using water (e.g., cleaning) due to residual moisture [22].

*Other process parameters*

The antimicrobial efficiency of a CAP also depends on several other process parameters, microbial factors, and food characteristics. This includes the kind of discharge system, the exposure time, process gas (oxygen and water content), and its flow rate [73,76]. Other factors are the type and amount of reactive species [35] and applied electrical properties (power, voltage, and frequency) [67]. The presence of UV radiation and the mode of application (direct or indirect), i.e., the distance between the sample- and the plasma-emitting unit, play a major role [76]. In general, higher energy input increases microbiological inactivation due to increased generation and concentration of reactive species [67]. The exposure time consisting of treatment time and retention time is crucial for determining the damage to the cells and spores [69]. In open systems, the treatment time is key, but in closed systems, such as in-package applications, the retention time often exceeds treatment time, thereby potentially enhancing the antimicrobial effect until the reactive species final decay [73,77]. Furthermore, the latter mitigates the risk of recontamination. Multiple, single, or short-interval applications show different results, where repeated exposure is more detrimental for the microorganisms [39].

In general, oxygen-containing process gas mixes show better antimicrobial effects than oxygen-free gas mixtures under identical process conditions [50]. Etching caused by ROS is the most relevant antimicrobial process, which can also lead to weight loss of the product due to the removal of top surface layers [40,78].

*Product characteristics*

CAP treatment is influenced by the product’s physical state, pH, water content, surface characteristics (e.g., porosity, roughness), and composition [38]. Rough and porous surfaces can deteriorate the antimicrobial effects by sheltering microorganisms from CAP. Stomata, rifts, cuts, pits, etc., facilitate bacterial adhesion and might enable migration into the plant tissues [24,76]. Smooth surfaces (like tomatoes) can be decontaminated more easily than rough complex surfaces [24], such as barley grains. Waxy components on the surface can provide protection from reactive species or facilitate adherence of microorganisms [29]. Generally, most microbial contamination is located in the outer layers of the pericarp [79].

*Microbiological resilience*

The resilience to CAP is influenced by type, strain, morphology (spore or vegetative), concentration and physiological state, surface complexity, occurrence (natural or inoculated), and the presence of other microbes or in biofilm [23,24,69,73]. Spores are more resistant than vegetative cells, and bacteria are more sensitive than fungi, which might be linked to structural and compositional differences between prokaryotic and eukaryotic cells [67]. Some microorganisms are more sensitive to UV radiation, while others are more vulnerable to oxidative stress [24]. Pigments like melanin protect them from cytotoxic agents like UV radiation [80]. High initial microbial counts and the presence of multiple microbial layers increase treatment time. The top layers can act as a physical barrier protecting the layers underneath from reactive species [71] or UV radiation, mitigating the decontamination effect [23]. The same occurs if microorganisms hide in the stoma of plants or rifts of grains [76]. However, longer CAP exposure increases decontamination, but can reduce germination capability and growth potential of grains [81], sometimes due to heat [75].

Several studies found that native microbiota are more resistant than inoculated microorganisms [51,75,81]. Native microbiota are more embedded and, therefore, less exposed to the plasma species than inoculated ones [75]. Autoclaving grains before inoculation possibly affect their surface conditions and influence microbiological inactivation.

*Inactivation kinetics*

CAP inactivation kinetics start with a rapid decline in microorganisms because of accessibility for reactive species and UV radiation. The following phase is slower due to the protection of inner biofilm layers and cells by upper layers of dead cells and debris, which first have to be eroded before being reached [68,69]. Similar shelter effects by stoma or rift can influence the inactivation kinetics [76]. During CAP exposure to mixed microbiota, more vulnerable microorganisms might be inactivated in the initial phase, while more resilient microbes or durable forms like spores are inactivated at a later stage.

Consequently, the antimicrobial CAP efficiency depends on several different product, microbiological, and process parameters that are often not easy to control. Thus, the outcome is difficult to estimate beforehand and not easy to evaluate during the process, which makes process control challenging. Different studies used different setups and parameters that make a comparison complicated or even impossible. Hence, a CAP treatment has to be verified on a case-by-case basis, i.e., a particular CAP system setup for a specific product and process goal. Therefore, the following two sections are going to focus in more detail on the CAP effects on bacteria and fungi present in barley grains.

### 4.1. CAP Effects on Bacteria

Bacteria can reduce the quality and quantity of yield but are usually less severe than fungi [23]. CAP often shows a stronger effect against bacteria than fungi [80]. Thus, CAP effects on bacteria present on grain are less studied [23]. Therefore, the following subsection will start with the general CAP effect on bacteria and close with specific data on barley grains.

*Bacterial cells*

CAP treatment can have lethal, sublethal (bacteriostatic impact), or nonlethal effects (metabolic alterations) on bacteria [37]. It can lead to substantial morphological and structural changes like a high degree of electroporation, breaking of membrane, deformation, and leakage of cytoplasm. These alterations are often linked to ROS, UV radiation, the electric field, and charged particles [69]. The reaction dynamics between ROS (i.e., OH^•^, H_2_O_2_, O^•^, and O_3_) and structural bonds of peptidoglycan (i.e., C−O, C−N, or C−C bonds) that form bacterial cell walls show different efficiency with H_2_O_2_ as the least effective [82]. This underlines the crucial role of ROS for bacterial inactivation by CAP, causing cell wall injuries and possibly cell cleavage [12,83]. Generally, aerobic bacteria are more resistant to inactivation by ROS than anaerobes [37]. But bacterial inactivation rates of air-based CAP can be boosted by process parameters increasing the generation of ROS [24]. Other inactivation mechanisms include the build-up of charges on the cell surface, leading to electroporation, but also mechanical destruction caused by energetic plasma particles [35].

Gram-negative bacteria are more rapidly inactivated by CAP than Gram-positive cultures, also when present in biofilms or as planktonic cells [83,84]. This is linked to the thinner, less protective outer membrane of Gram-negative bacteria [71] made of phospholipids and lipopolysaccharides [83]. Those lipids are most vulnerable to oxidative stress, and membrane penetration of reactive species is more likely due to the occurrence of pore-forming proteins, often causing cell leakage [83]. On the contrary, the cell envelope of Gram-positive bacteria is made of thick layers of peptidoglycan that can withstand ROS attacks and cause no cell leakage [69]. Here, certain reactive species like singlet oxygen or hydrogen peroxide migrate into the cell without affecting its wall or membrane [38]. They are inactivated by the intercellular breakdown of proteins essential for cell function, such as DNA and enzymes [35,38,69]. However, in some cases, Gram-negative bacteria are more resistant, indicating that CAP sensitivity does not only depend on cell wall properties [83]. Cell-supporting media could mitigate the inactivation process [69,75].

Bacterial cell shape can influence CAP inactivation; e.g., cocci are more resistant than rod-shaped cells [74]. Electrostatic charges are more likely to inactivate nonspherical cells by interrupting the surface charge equilibrium than cocci without mechanical breakdown of the cell wall [37]. This mechanism can be facilitated by a higher surface roughness in Gram-negative bacteria due to the outer membrane itself [83]. Surviving bacteria cells showed reduced metabolic activity, i.e., they are in a viable-but-nonculturable state [74]. Their genes managing housekeeping and ion transport are downregulated, but their response to DNA repair processes is elevated [35]. Bacteria can recover, but no stress hardening or resistance-building effect was observed [35]. The bacterial growth phase (steady or logarithmic) has little to no impact on CAP sensitivity [67].

*Bacterial spores*

Bacterial spores possess a higher tolerance against external stress, including CAP, due to their complex structure, low water content [73], high concentration of dipicolinic acid [38,67], and inactive metabolism [37]. CAP treatment can damage or break the spores’ membranes and degrade enzymes, proteins, and dipicolinic acid, thereby inhibiting spore germination [25,69,71]. Hence, key inactivation mechanisms of the reactive species and UV radiation are comparable to the processes during bacterial inactivation [25].

*Planktonic and biofilm cells*

Microorganisms often form biofilms for their growth, while the planktonic mode is mainly used for spreading [76]. Bacterial biofilms grow on different substrata, including food and food contact surfaces, thereby creating a source for recurring contamination [74]. These sessile communities of bacteria cells are surrounded by a matrix of extracellular polymeric substances (EPSs) [73]. EPSs consist of polysaccharides, lipids, proteins, and nucleic acids, which can react with plasma species, thereby protecting the embedded bacteria [74,83]. The biofilm composition varies with regard to the type and strain of bacteria as well as the surface medium to which the microorganism is attached [74]. CAP effects on biofilms are the decomposition of EPSs, the decrease in biofilm thickness, damage to the cells, lower culturability, and reduced cell metabolism [73]. The cross-protection phenomenon can reduce CAP efficacy, i.e., biofilms formed under stress conditions, e.g., cold stress, showed higher resistances than biofilms grown under non-stress circumstances [36]. Process parameters (e.g., energy input, gas, etc.) define the penetration depth of plasma species into biofilm, which reaches the cells via the water channels [26]. Usually, not all bacteria in biofilms are inactive by CAP, but their growth is typically reduced [37]. Single-species biofilms showed a higher CAP sensitivity than biofilms of mixed microflora [83]. Protective layers, e.g., inactivated bacteria on top or soiling (in agricultural produce), protect the biofilm underneath reducing the decontamination efficacy [29,68].

CAP affects microorganisms differently, which might change the composition of background microflora on the grain. Their interactions play a key role in grain quality. For example, *Bacillus* spp. can curb the growth of *Fusarium* and facilitate plant growth. CAP application for 5 min resulted in changing the relative abundance of *Bacilli* from 45% to 80% after direct treatment and 93% after indirect treatment, respectively, making it the most prevalent species [85]. The following Table 1 shows the maximal bacterial reduction by CAP with regard to barley grains.

The CAP treatment of barley grains using air plasma showed increased bacterial inactivation rates with longer treatment and retention time. The reduction was higher during direct exposure, but a complete inactivation was never accomplished. Spores showed more resilience than vegetative cells, and background microbiota were more resilient than inoculated microorganisms. Interestingly, the background microbiota of wheat were more susceptible to the identical CAP treatment than the one of barley [51]. Argon plasma reduces bacterial spores less effective despite constant shaking and temperatures up to 50 °C during exposure [86]. This finding underlines the need for oxygen in the process gas to optimise bacterial inactivation. In most cases, one cultured species was inoculated on autoclaved seeds, which does not reproduce natural living conditions and the occurrence of bacteria. This might have influenced the outcome of the experiments not showing the actual decontamination potential. Most bacteria live in multi-species environments, interacting among each other to enhance metabolic exchanges and survival. Thus, results concerning inoculated bacteria might have a reduced significance for practical application, because the overestimate the CAP efficiency.

In any case, the industrial-scale CAP treatment of barley grains has to be performed under dry conditions [16,17], where the proliferation of bacteria and their presence as vegetative cells, including biofilms, is not an issue due to the practical absence of water. Potential further research should use air as a process gas due to its cost advantages and the presence of oxygen. It should concentrate on the inactivation of naturally occurring bacterial spores due to a lack of studies and assumed resilience. Still, CAP application can be a useful and effective tool for bacterial decontamination of barley grain.

### 4.2. CAP Effects on Fungi and Mycotoxins

The presence of fungi and their mycotoxins reduce yield and profit [23,47]. The inactivation of fungi also mitigates the threat of mycotoxin production [72]. CAP treatment using air as process gas can be used for reducing fungal contamination of grain and destroying their mycotoxins. Therefore, the following subsection will start with general CAP effects on fungi including its mycotoxins and close with specific data on barley grains.

*Fungal cells*

Fungi, as eukaryotic organisms, possess additional cellular protection mechanisms against oxidation or DNA damage than bacteria and often have a favourable (i.e., lower) surface-to-volume ratio, which benefits the dispersion of reactive species [22]. During CAP exposure, fungal membranes are etched or oxidised by reactive species or damaged due to the accumulation of charged particles [35]. Ozone can compromise membrane integrity by peroxidation of its phospholipids, which increases permeability [34,87]. In addition, electrostatic forces can exceed the membrane’s tensile strength, leading to breakage [35] and, subsequently, deformation and leakage of the fungal cells [72]. ROS entering the cells can alter proteins, leading to the loss of their function for the signalling or transport of metabolites [35]. Irreparable DNA damage like crosslinks or lesions stops their replication and transcription, finally causing death [35]. UV radiation can also alter fungal cells [35], with the UV-C spectrum showing the strongest biocidal effects on fungal DNA and its cell walls [72]. Fungi cell walls might contain pigments [80], which improve their resistance [67].

*Fungal spores*

Fungal spores are more resistant to CAP treatment than vegetative cells [67]. Their dark pigmentation increases resistance against UV radiation compared to other spore-forming microorganisms, which prevents damage to DNA and proteins [71]. They require higher UV doses for inactivation but are still sensitive to oxidation [32]. Natural background fungi generally showed higher resilience than inoculated spores regardless of application mode (direct or indirect) and treatment or retention time [51]. The inactivation of fungal spores is linked to cell wall destruction and cell leakage [67].

*Mycotoxins*

Mycotoxins possess various noxious effects and are often colocated in the same samples [3,88], which can increase their toxicity [89], setting in at low concentrations [72]. Their breakdown by plasma in solutions is a lot faster than on solid food surfaces due to better accessibility of the molecules by reactive species and an occurring hurdle effect, i.e., the combination of reactive species and low pH. In addition, the food matrix can react with reactive species, thereby reducing the CAP efficiency [34]. The degradation of mycotoxins by air plasma is influenced by their chemical structure but not affected by their molecular mass [90]. Mycotoxin molecules with long aliphatic chains (fumonisin B_1_ and AAL-toxin) degrade fast, while molecules with a firm structure of condensed aromatic rings (like sterigmatocystin) take longer to decay [90,91]. Compounds with a mixed structure have intermediate half-lives [90]. Elevating oxygen and humidity levels can enhance mycotoxin degradation due to larger concentrations of hydroxyl radicals and ozone [34], resulting in non-toxic or lower toxic compounds [92]. However, this was not always observed in CAP-treated barley grains [53]. The breakdown pathway is mainly linked to oxidation, including succeeding addition reactions (of water molecules, hydrogen atoms, etc.) and also cleavage reactions [34]. It can be further increased by the presence of UV radiation [34], resulting in the case of aflatoxins in structural changes to their furan ring together with the cleavage of their lactone moiety [72]. Ozone degrades various mycotoxins present on the grains while barely affecting their product qualities [72,93]. An electrophilic attack of ozone on unsaturated compounds in mycotoxin molecules alters and degrades them [94].

Table 2 below shows the maximum reduction rates of the fungi and mycotoxins on barley grain due to CAP application.

Comparing the above-mentioned results is difficult due to different experimental setups, including target organism/mycotoxins and few studies available. Nevertheless, DBD systems using air as a process gas have been shown under various settings to successfully reduce fungi and their mycotoxins present in barley grains. The CAP efficiency mainly depends on plasma chemistry, exposure time, mode of CAP application, fungal strain and mycotoxin. The inactivation mechanisms of fungal cells by CAP were less severe but comparable to those of bacteria.

Mycotoxin degradation is a complex process depending on the combined reactions of different plasma products. Its efficiency is determined by molecule structures and chemical bond forces of the treated analyte, which define its chemical conversion rate into other (less toxic) compounds. A complete detoxification of barley grains was never achieved. CAP effects on solid products are limited to the surface penetration depth of the plasma constituents. They might be scavenged by reactions with the treated product or fungi cell, protecting the mycotoxin. In rare cases, the fungal reduction was not associated with the decrease in its mycotoxins [50]. Further research on this phenomenon is essential for setting process parameters correctly and anticipating the desired outcome correctly.

Pretreatment of barley grains with CAP reduces the required amount of the chemical fungicide Vitavax 2000 (Chemtura Co., Bratislava, Slovakia) but still achieves the same decontamination effect [47]. This synergy was attributed to enhanced wettability and adhesion of the chemical to barley grains. Further research is needed to confirm this for other pesticides.

## 5. CAP Effects on Storage Insect Pests

More than 100 potential insect species are known to infest barley at different stages of its production cycle [2]. The ongoing climate change already led to biodiversity loss and an increase in certain insect pests on the field [96]. These infestations contribute to deteriorating grain quality during storage if not detected or handled properly [97]. Insects transmit fungal spores and facilitate mould growth, including mycotoxin generation, due to their metabolic activities creating heat and moisture in the stock [45,98]. CAP treatment using air as process gas can disinfest barley grains due to its high insecticidal effect, which is realised in a few minutes compared to several minutes for UV radiation and hours if solely ozone is applied [52]. Therefore, the following subsection provides an overview of CAP effects on insect pests found on barley grains.

*Insecticidal effects*

CAP’s main insecticidal mechanism is linked to ROS that enters the insects’ body via the spiracles and trachea, resulting in lethal cell alterations and affecting the neuromuscular system [56]. The activity of endogenous growth enzymes is also impeded [99]. Furthermore, their vulnerability and inactivation depend on the life stage. Eggs are killed by ROS damage to the embryos and oxygen deficiency, impeding hatching. Larvae are inactivated by oxidative stress caused by ROS, injuring their haemolymph and skin, leading to body deformation. Adult insects are inactivated by damage to their nervous system in combination with anoxia conditions, but also due to oxidation to their exoskeleton [70].

The ozone plays a major role in inactivating insects. Their mortality rate depends on saturation time (i.e., ozone concentration at the beginning of treatment), decomposition kinetics of ozone (which is influenced by humidity), exposure time, insect species, and life stage, but not on temperature [43,45]. Insect inactivation with ozone alone usually takes several hours or even days but does not alter the grain quality or leave any residues. This makes additional aeration for its removal compared to other fumigants obsolete [43]. Ozone exposure to insects decreases their metabolism and causes death due to low oxygen availability and oxidative stress on a cellular level, i.e., oxidation of lipids, proteins, and DNA. However, eggs and pupae possess a lower respiration rate and protective outer layers, making them often less vulnerable to ozone treatment than adult insects [45]. Moreover, grains can provide shelter and react with ozone, decreasing their insecticidal efficiency [43]. Higher ozone concentrations reduce the reproduction of surviving insects but also decrease their mobility and velocity in grain storage [45].

The most prevalent insect pests in grain storage are beetles of the species *Tribolium castaneum* and *Tribolium granarium*. Their mortality rates significantly increase with increasing voltage and treatment time, along with reduced electrode distance during direct exposure [100,101,102]. The inactivation rates are influenced by the mode of application, developmental stage, and post-treatment retention time [103]. Direct treatment is most effective regardless of post-treatment storage, and adults show the most resistance to it compared to eggs, young and older larvae, or pupae. Indirect treatment is generally less effective for all life stages, but long post-treatment storage often significantly enhances inactivation rates further, frequently reaching 100% [103].

Insect CAP mortality is affected by the synergy effects of ROS together with temperature (40 to 45 °C), highly energetic electrons, and electromagnetic field [102]. The intercellular presence of ROS leads to lipid peroxidation growing with treatment times and activation of antioxidative enzymes [103], which is a protective mechanism of insect cells [102]. Total inactivation of *T. castaneum* can be achieved in different life stages (egg, larva, and adult), but this requires long plasma exposure and strength [52,100]. A significant reduction is often possible even if present grains mitigate the CAP effect, preventing a complete inactivation. However, larvae can show higher CAP resistance due to the presence of long, dense hairs, which reduces direct body contact with the plasma [102]. In addition, lower respiration rates and different morphological characteristics (e.g., harder or softer cuticles or the presence of waxes) at different life stages could make the insects less vulnerable [103].

Surviving insects suffer permanent injuries, e.g., they show limited responses to stimuli, or the larvae develop into pupae, but no hatch occurs or is delayed [52,100]. Insects exposed to sublethal CAP levels have a significantly lower weight and respiration rate, which can mitigate the uptake of toxic gases and reduce their metabolism [103]. Their cuticle, epidermis, and fat bodies can be damaged by CAP, which triggers further immune response and complex biochemical modifications inside the insects, i.e., clotting and melanisation [102,103]. The removal of cuticular lipid layers increases water losses [99], which causes darker colour, physiological changes, or even death [52,103].

Comparing the above-mentioned, conducting studies is difficult due to different setups, process parameters, sometimes missing information (e.g., used process gas) or deviating definitions. For example, mortality rates were not identically defined, sometimes including surviving but injured individuals. Generally, CAP shows better insecticidal effects during direct than indirect treatment, regardless of their life stage. But those results were achieved after several minutes and in narrow gaps, which is inapplicable in industrial scale applications for large grain volumes. Post-treatment retention increases the inactivation efficacy, but the presence of grains and grain material mitigates this effect. All life stages showed slightly different CAP mortalities, most likely due to their differing respiration rates and different morphological characteristics (e.g., harder or softer cuticles or the presence of waxes). Inactivation by air plasma was mainly attributed to ROS. Reactive species damage the insects’ nervous system and cuticular layers, which can prevent further development (ecdysis) or remove the protective waxy film, leading to dehydration and possibly death. Surviving insects show abnormal behaviour or apathy, possibly due to an impaired nervous system. Oxidative degradation of haemolymph and other body fluids like lipids negatively affects their body functions and reduces mobility. Little research on storage insect pests has been conducted so far. So, little is known about the CAP effects, such as UV radiation and RNS, on those species.

## 6. CAP Effects on Seed Dormancy, Germination, and Plant Growth

Seed dormancy is crucial for plants in their life cycle [104], i.e., choosing the most suitable conditions for germination and subsequent growth. Germination is the process of the radicle [35,105] or coleoptile [46] emerging from the seed. It requires optimal oxygen, water, and temperature conditions but can also be affected by light and nitrate [49]. The germination of barley grain is important for sowing and malt production. CAP can be used for priming germination as an alternative to conventional techniques [35] as it enhances seed germination [46], vigour of the seedling [47], plant growth [23], and also seedling mass [65] in barley grains.

*Barley grain dormancy*

The antagonistic plant hormones abscisic acid (ABA) and gibberellic acid (GA) control seed dormancy [104]. Their content is managed by biosynthetic and degradation enzymes [106]. In barley, the ABA content of the embryo induces and maintains dormancy. It is sensitive to oxidation, but the pericarp and seed coat decrease its oxygen availability together with scavenging phenolic matter [107]. Barley grains metabolise ROS during germination to manage GA/ABA levels [104]. An antioxidative mechanism in seeds balances the degree of oxidisation to meet the “oxidative window” for germination, which uses catalase, for example [104].

*Breaking dormancy*

CAP application can be used for seed priming, resulting in an improved germination rate, germination yield [56], and more uniform plant growth [108]. Seed germination starts with water imbibition through seed testa [30]. Pristine seeds possess a hydrophobic, waxy outer layer that is eroded by CAP, making them permeable to water [46]. This facilitates potential higher water uptake [23]. In addition, etching or eroding processes can leave cracks in the bran [35] or thin the seed coat, making the uptake of oxygen easier [108]. Also, ROS or H_2_O_2_ can enter the grain more easily and reduce the plant hormone ABA by oxidation [109] or trigger cell-inherent ABA catabolism processes [104]. These physiochemical changes influence the synthesis of plant hormones [23,70], including the GA [104]. GA breaks seed dormancy by inducing unique gene expression [30], e.g., the production of hydrolytic enzymes like α-amylase [65]. The production of GA increases with enhanced water uptake [30,65]. These catabolic processes initiated by CAP cause higher sugar and lower starch contents in treated grains that can accelerate the germination of barley [58,110]. However, ROS might also react with or influence the synthesis of other phytohormones, signalling molecules, or inhibitors (e.g., antioxidant enzymes) that affect the dormancy and growth of barley [104].

However, a decreased ABA content is not always associated with an improved germination rate. When comparing non-dormant and dormant barley seeds, only the latter showed a positive effect [104]. Direct CAP treatment of dry barley seeds shows the strongest stimulating effects, followed by indirect treatment using plasma-activated water, but the direct treatment of water-soaked grains completely inhibits germination [110]. The effect of direct DBD plasma shows enhanced germination rates, especially in the first 24 h, but after 72 h, no big difference can be observed [46]. Direct CAP application can also improve other parameters like seedling vigour, but excessive treatment has adverse effects on all germination parameters [47,58,110]. This includes average root diameter, root volume, and germination percentage [53]. The optimal time settings can range from seconds to minutes depending on plasma characteristics and the dormancy status of seeds [65]. The CAP effect on germination parameters of barley grains is presented in Table 3.

However, the stimulating effects on germination rates and growth parameters continuously deteriorate over time until they disappear [56,105]. This includes water permeability, which only lasts a few weeks [46].

*RNS as nutrients*

RNS provide easily absorbable forms of nitrogen to the grain that can act as fertilisers or stimulants [70]. Optimal CAP treatment can increase the total contents of soluble sugars, amino acids, and phytochemicals like γ-aminobutyric acid within the sprouts, which leads to faster growth of barley [111]. Physical conditions of sprouts can be enhanced too, i.e., longer hypocotyl length and much denser and longer roots. However, these effects were not often remarkably different from control [30].

*Stress hardening*

Stress conditions, e.g., drought, higher soil salinity, and low temperature, can cause hormonal and metabolic imbalances in the barley seeds, which adversely affects the germination process [49]. CAP can trigger intrinsic mechanisms for stress response [48], because the germination process is not only regulated by hormones but also RNS and ROS [49]. Its positive effects on barley include an increased content of pigments, stimulated enzyme activity, and an enhanced root system that facilitates the uptake capacity of nutrients during the initial stages of plant growth [48,49].

*Decontamination vs. germination*

CAP can be used for the decontamination of seeds without negatively affecting germination [112]. However, CAP conditions used for achieving optimal germination rates are often shorter and milder than settings required for microbiological decontamination [65,75] or disinfestation [102]. Excessive CAP exposure closes the “oxidative window” for germination due to oxidative damage [104] and decreases the germination rate [23].

CAP treatment of barley grains shows promising results in boosting germination, providing aid for seedlings and in early plant development also under stress conditions. This is attributed to synergetic actions, including surface decontamination, increased wettability, activation of enzymes, and additional nutrients provided by RNS due to fixed nitrogen. Those positive effects depend on several process parameters but are limited to short exposures and are further influenced by the seeds’ dormancy status. Unfortunately, the available studies did not evaluate the later stages of plant development or the final crop yield of CAP-treated barley grains. However, the CAP effect seems to be limited to the early stages of plant growth.

In practical terms, CAP could speed up malting processes that result in germ reduced, healthier products. Furthermore, nitrogen-based stimulants generated by CAP could be an advantage in organic farming due to their pure physical nature and chemical-free character.

## 7. Conclusions

As a novel and versatile technology, CAP can be used as a dry process for plenty of different applications, as shown above. Its generation does not require water or chemicals. Due to its operation at low temperatures and short time span, little energy is consumed, and additional production steps like cooling can be avoided. CAP shows stronger effects during direct than indirect treatment regardless of the used DBD plasma source, but exposure to DCSBD delivers the fastest results. However, the efficacy of indirect exposure can be increased by longer post-treatment retention times. ROS were identified as the most relevant reactive species due to their strong oxidative effects for killing insect pests, inactivating microorganisms, destroying mycotoxins or breaking grain dormancy.

However, CAP is not a one-size-fits-all solution. Short treatment and milder process parameters can facilitate germination and plant growth but barely decontaminate or disinfest the grains. Stronger CAP applications might reduce microorganisms and insects but deteriorate germination rates. Complete inactivation of pests and mycotoxins on grains by CAP alone was never achieved, most likely due to the limited penetration depth of the plasma constituents and the mitigating effect caused by their reactions with grain material. But a combined treatment of CAP and fungicide showed synergetic effects fully removing the inoculated fungus while using only 10% of the recommended dose [47].

Process parameters are often not easy to control (e.g., fluctuating air humidity). This can noticeably influence CAP chemistry and its final result. Therefore, the outcome is difficult to estimate beforehand and not easy to evaluate during the process, which makes process control challenging. Currently, the result has to be evaluated on a case-by-case basis, i.e., a particular CAP system setup for a specific product and purpose. Establishing standards for CAP technology in the food and agriculture sector could help provide constant, reproducible product quality. This could also make the outcome more predictable and help untrained personnel become productive in a shorter time. In addition, an indicator organism for verifying the outcome of a CAP process should be selected from naturally occurring background fungi. They showed the strongest CAP resistance and pose the highest health risks due to potential mycotoxin production.

## 8. Future Directions

Many studies provide important insights, but there is still a need for further research. Native background microbiota showed the strongest resistance to CAP and more research should be conducted in this regard. They have mutualistic interactions with plants and potentially inhibit the growth of harmful species [85]. Depending on their individual sensitivity, CAP can change their relative abundance. This might influence future product quality. However, studies mostly used cultured and inoculated species, which do not mimic natural conditions. Results on their inactivation appear stronger and might have reduced significance for practical application.

Research on storage insect pests should focus on indirect CAP applications, which are technically similar to existing fumigation practices, e.g., in silos [45,113]. Additional findings on insecticidal RNS effects might help optimising process parameters for insect control. And lastly, a combined application of CAP and pesticides showed synergetic effects. Further research in this field can help overcome the decontamination limitations of CAP and reduce chemical use as required by the European Green Deal.

## Figures and Tables

**Table 1:** Bacterial decontamination of barley grains by CAP treatment.

Bacteria	Plasma Characteristics	Treatment Time	Maximum Reduction	Reference
Background bacteria	Closed DBD system; 80 kV; air	20 min (+24 h storage)	2.4 log (direct) ≈52.2% reduction; 1.7 log (indirect) ≈37.0% reduction	[51]
*Escherichia coli* (inoculated)	Closed DBD system; 80 kV; air	20 min (+24 h storage)	3.5 log (direct) ≈97.7% reduction; 3.3 log (indirect) ≈91.7% reduction	[51]
*Bacillus atrophaeus* (vegetative cells; inoculated)	Closed DBD system; 80 kV; air	20 min (+24 h storage)	3.2 log (direct) ≈66.7% reduction; 2.7 log (indirect) ≈56.3% reduction	[51]
*Bacillus atrophaeus* (endospores; inoculated)	Closed DBD system; 80 kV; air	20 min (+2 h storage)	2.4 log (direct) ≈33.4% reduction; 1.3 log (indirect) ≈16.7% reduction	[51]
*Bacillus atrophaeus* (endospores; inoculated)	Volume-DBD; 12 kV; argon	2 × 5 min with 5 min break in between	1.43 ± 1.38 log (direct) ≈19.3% reduction	[86]

**Table 2:** Fungal and mycotoxin decontamination of barley grains by CAP treatment.

Fungus/Mycotoxin	Plasma Characteristics	Treatment Time	Maximum Reduction	Drawback	Reference
*Fusarium culmorum* (inoculated)	Diffuse Coplanar Surface Barrier Discharge (DCSBD); 14 kHz, 20 kV, 400 W; ambient air	120 s	Complete fungal removal	Germination and seedling vigour reduced after >30 s	[47]
*Aspergillus niger* (inoculated)	DCSBD; 350 W; dry air	180 s (+14 d storage at 9 °C)	3 log ≈43.6% reduction (mycelium); 2.5 log ≈47.9% reduction (spores)	Certain storage and incubation conditions might facilitate mycotoxin production	[50]
*Penicillium verrucosum* (inoculated)	DCSBD; 350 W; dry air	180 s (+14 d storage at 9 °C)	3.12 log ≈55.5% reduction	Certain storage and incubation conditions might facilitate mycotoxin production	[50]
Zearalenone (inoculated)	DBD (in pulsed mode); 3.5 kHz, 0–30 kV; air	60 s 180 s	52.7% 64.8%		[95]
Deoxynivalenol (inoculated)	Direct DBD (in pulsed mode); 3.5 kHz, 0–34 kV, 300 W; air	6 min 10 min	48.9% 54.4%	No significant increase in toxin degradation due to increased humidity and moisture	[53]
*Penicillium verrucosum* (spores; inoculated)	Closed DBD system; 80 kV; air	20 min (+24 h storage)	3.6 log (direct) ≈56.3% reduction; 2.7 log (indirect) ≈42.2% reduction		[51]
Background fungi	Closed DBD system; 80 kV; air	20 min (+24 h storage)	2.1 log (direct) ≈47.7% reduction; 1.5 log (indirect) ≈34.1% reduction		[51]

**Table 3:** DBD plasma effects on germination of barley grains.

Plasma Source	Gas	Power, Voltage, and Frequency	Time	Germination Rate	Reference
Diffuse Coplanar Surface Barrier Discharge (DCSBD)	Ambient air	400 W, 20 kV, 14 kHz	control 15 s 30 s 60 s 120 s	100% 100% 98 ± 4% 94 ± 8% 72 ± 15%	[47]
DBD (in pulsed mode)	Air	3.5 kHz, 0–34 kV, 300 W	control 6 min	80% 93.3%	[53]
DBD (direct)	Air	1 kHz, 8.6 kV, 5.3 W	control 3 min	92% 94–100%	[46]
DBD (direct)	Ambient air	50 Hz, 16 kV	control 5 min	42% 78% in 7 days	[110]

## Data Availability

No new data were created or analysed in this study. Data sharing is not applicable to this article.
